# The clinical utility of biomarkers in diagnosing Major Depressive Disorder in adults: A Systematic Review of literature from 2013 to 2023

**DOI:** 10.1192/j.eurpsy.2025.793

**Published:** 2025-08-26

**Authors:** S. H. Ang, R. Ho Chun Man, R. S. McIntyre, S. K. Chiang, K. M. Teopiz, Z. Zhang, C. Ho Su Hui

**Affiliations:** 1 Yong Loo Lin School of Medicine, National University of Singapore, Singapore, Singapore; 2 Department of Psychological Medicine, Yong Loo Lin School of Medicine, National University of Singapore; 3 Department of Psychological Medicine, National University Hospital, Singapore, Singapore; 4 Department of Psychiatry, University of Toronto; 5 Mood Disorders Psychopharmacology Unit, Univesity Health Network; 6 Brain and Cognitive Discovery Foundation, Toronto, Canada; 7 Anhui Engineering Research Center for Intelligent Computing and Application on Cognitive Behavior, Anhui, China

## Abstract

**Introduction:**

The variety and efficacy of biomarkers available that may be used objectively to diagnose Major Depressive Disorder (MDD) in adults are unclear. This systematic review aims to identify and evaluate the variety of objective markers used to diagnose MDD in adults.

**Objectives:**

This systematic review aims to identify and evaluate the variety of objective markers used to diagnose MDD in adults.

**Methods:**

The search strategy was applied via PubMed and PsycINFO over the past 10 years (2013-2023) to capture the latest available evidence supporting the use of biomarkers to diagnose MDD. Papers were excluded if they were published in a non-peer-reviewed journal and/or not published in English; featured non-primary study designs (e.g. systematic review, meta-analysis, literature review); included children or adolescents in the study population; featured participants without a clinical diagnosis of MDD; featured participants with a diagnosis of other forms of MDD such as treatment resistant depression, vascular depression, remitted depression. Data was reported through narrative synthesis.

**Results:**

42 studies were included in the review. Findings were synthesised based on the following measures: blood, neuroimaging/neurophysiology, urine, dermatological, auditory, vocal, cerebrospinal fluid and combinatory – and evaluated based on its sensitivity/specificity and area under the curve (AUC) values. The best predictors of blood (MYT1 gene), neuroimaging/neurophysiological (5-HT1A auto-receptor binding in the dorsal and median raphe), urinary (combined albumin, AMBP, HSPB, APOA1), cerebrospinal fluid-based (neuron specific enolase, microRNA) biomarkers were found to be closely linked to the pathophysiology of MDD.

**Conclusions:**

A large variety of biomarkers were available to diagnose MDD, with the best performing biomarkers intrinsically related to the pathophysiology of MDD. Potential for future research lies in investigating the joint sensitivity of the best performing biomarkers identified via machine learning methods and establishing the causal effect between these biomarkers and MDD.

**Disclosure of Interest:**

None Declared

